# Influence of Surgeon Experience and Clinic Volume on Subjective Knee Function and Revision Rates in Primary ACL Reconstruction: A Study from the Swedish National Knee Ligament Registry

**DOI:** 10.1177/23259671241233695

**Published:** 2024-03-11

**Authors:** Dzan Rizvanovic, Markus Waldén, Magnus Forssblad, Anders Stålman

**Affiliations:** † Department of Molecular Medicine and Surgery, Stockholm Sports Trauma Research Center, Karolinska Institutet, Stockholm, Sweden; ‡ Department of Orthopaedics, Växjö Central Hospital, Region Kronoberg, Växjö, Sweden; § Unit of Public Health, Department of Health, Medicine and Caring Sciences, Linköping University, Linköping, Sweden; ‖ Capio Ortho Center Skåne, Malmö, Sweden; ¶ Ortopedi Stockholm, Stockholm, Sweden; # Capio Artro Clinic, Sophiahemmet, Stockholm, Sweden; Investigation performed at Stockholm Sports Trauma Research Center, Karolinska Institutet, Stockholm, Sweden

**Keywords:** ACL, ACLR, patient-reported outcome, revision rate, surgical volume

## Abstract

**Background:**

Anterior cruciate ligament reconstruction (ACLR) performed by high-volume surgeons/clinics has been associated with increased graft individualization and decreased operating times, complication rates, and total costs.

**Purpose:**

To investigate the influence of surgeon/clinic volume on subjective knee function and revision surgery rates at 2 years after primary ACLR.

**Study Design:**

Cohort study; Level of evidence, 3.

**Methods:**

Data from the Swedish National Knee Ligament Registry were used to study patients who underwent primary ACLR between 2008 and 2019. Surgeons/clinics were categorized based on a combination of total caseload volume (cutoff: 50 ACLRs/surgeon, 500 ACLRs/clinic) and annual volume (cutoff: 29 ACLRs/year/surgeon, 56 ACLRs/year/clinic). The thresholds of minimal important change (MIC), Patient Acceptable Symptom State (PASS), and treatment failure (TF) relative to the Knee injury and Osteoarthritis Outcome Score (KOOS) and KOOS_4_ (mean score of the KOOS Pain, Symptoms, Sports/Rec, and QoL subscales) were applied. Adjusted multivariable logistic regression was performed to assess variables influencing the MIC, PASS, or TF of the KOOS and KOOS_4_. Adjusted Cox regression analysis was conducted to determine the hazard ratio of subsequent ACLR.

**Results:**

Of 35,371 patients, 16,317 had 2-year follow-up outcome data and were included. Patients who underwent primary ACLR by high-volume surgeons had significantly higher MIC and PASS rates and lower TF rates when compared with patients who underwent the procedure by low-volume surgeons: MIC_KOOS4_: 70.6% vs 66.3%; PASS_KOOS4_: 46.0% versus 38.3%; and TF_KOOS4_: 8.7% versus 11.8% (all *P* < .02). Significantly decreased odds of achieving MIC_KOOS4_ (OR, 0.74; 95% CI, 0.62-0.88) and PASS_KOOS4_ (OR, 0.71; 95% CI, 0.60-0.84) were found for ACLRs performed by low-volume surgeons. Clinic volume did not influence the odds of reaching MIC, PASS, or TF. Overall, 804 patients (2.3%) underwent subsequent ACLR at <2 years, with significantly higher revision rates among patients operated on at high-volume clinics (2.5% vs 1.7%; *P* < .001). However, in the adjusted Cox regression, surgeon/clinic volume had no influence on subsequent ACLR rates. High-volume surgeons/clinics had decreased time to surgery, operating time, perioperative complication rates, and use of thromboprophylaxis and nonroutine antibiotics (*P* < .001).

**Conclusion:**

Patients who underwent primary ACLR by high-volume surgeons experienced increased improvement and satisfaction regarding subjective knee function. Factors other than surgical volume influenced subsequent surgery rates. Patients might benefit from undergoing primary ACLR by high-volume providers.

Anterior cruciate ligament (ACL) reconstruction (ACLR) is a commonly performed surgical procedure in the United States and Europe.^25^ Despite extensive research within the field of ACLR, the literature on surgical volume and its influence on various outcomes related to ACLR is scarce. Previous studies have found that experienced surgeons perform ACLRs with shorter operating times,^4,18^ improved accuracy in terms of anatomic femoral tunnel placement,^12^ and an increased utilization of different autografts.^2,18,28^ In addition, patients undergoing ACLR by high-volume surgeons or at high-volume clinics have been shown to have a lower risk of readmission and lower total costs.^18,21,31^

However, to improve the management of ACL injuries, patient perspectives are important to emphasize when evaluating treatment outcomes. The Knee injury and Osteoarthritis Outcome Score (KOOS) is a commonly used patient-reported outcome measure in the Scandinavian knee ligament registries.^8,30^ KOOS results are typically presented as changes in mean or median scores or as absolute values, but these can be challenging to interpret clinically. To make the data more clinically relevant to patients’ experiences of improvement and to examine if acceptable results have been achieved after ACLR, 3 thresholds are commonly used: minimal important change (MIC), Patient Acceptable Symptom State (PASS), and patient-reported treatment failure (TF).^29^ The MIC is the smallest change in KOOS that patients consider meaningful and important, but even if a clinically relevant improvement is seen in MIC, it might not indicate whether patients consider themselves well.^14^ Therefore, the use of PASS helps to determine if patients have improved to a satisfactory level after ACLR.^13^ On the contrary, TF captures patients’ perceptions of failure, which is just as important as identifying treatment success.^13^

There is a paucity of literature on the influence of surgical volume on patient-reported outcomes after ACLR. A study using the Norwegian Knee Ligament Registry found that there was a higher proportion of subjective TFs among patients undergoing primary ACLR at low-volume clinics,^23^ whereas the relationship between surgeon experience and subjective knee function after ACLR has yet not been investigated. The rate of subsequent surgery is also important to consider when evaluating outcomes, as inconsistent results have been reported in various studies.^19,21,23,33^ Assessing surgical volume and its relationship to outcomes after ACLR is valuable, as it can provide novel insights to enhance surgical practices, improve patient outcomes, and guide informed decision-making.

Thus, the main purpose of this nationwide registry-based study was to investigate if surgeon and/or clinic volume affects subjective knee function (as measured by MIC, PASS, and TF for the KOOS) as well as to study the occurrence of subsequent revision surgery after primary ACLR among surgeons and clinics of different surgical volumes. Our hypotheses were that patients undergoing primary ACLR by experienced surgeons and at high-volume clinics would have better subjective knee function, in terms of improvement, satisfaction, and failures, as well as a lower revision rate at 2 years.

## Methods

A retrospective registry-based cohort study was conducted on prospectively collected data from the Swedish National Knee Ligament Registry (SNKLR). The SNKLR was established in 2005; it is a nationwide registry covering >90% of all the ACLRs performed annually.^16^ The SNKLR consists of both patient- and surgeon-reported data. Surgeons or other health care personnel at the clinics ask patients to fill in baseline data, such as height, weight, smoking status, and preoperative KOOS, while postoperative 1-, 2-, 5-, and 10-year follow-ups of KOOS are sent to them automatically. Surgeons report pre- and perioperative injury characteristics, such as cause of injury, associated knee injuries, intraoperative findings, surgical procedures, graft choice, perioperative complications, use of antibiotics and thromboprophylaxis, and duration of surgery. Patient characteristics (age and sex) are automatically collected through Swedish social security numbers. Subsequent surgeries (revision or contralateral ACLR) are reported separately and linked to the index ACLR.

Participation in this study was voluntary for both patients and surgeons. The study protocol received ethics review board approval, and the study was approved by the steering committee of the SNKLR.

### Study Population

The retrieved database included all primary (index and contralateral) and revision ACLRs registered in the SNKLR between 2005 and 2021. Patients with isolated ACL injury or with concomitant medial collateral ligament (MCL) injury undergoing primary index ACLR between January 1, 2008, and December 31, 2019, were further assessed for eligibility. Thus, all contralateral and revision surgeries were excluded. Patients who underwent surgery between 2005 and 2007 were also excluded since the total number of ACLRs per surgeon and clinic was unknown before the start of the registry, and therefore it was not possible to interpret the surgeon experience and clinic volume during the first registered years. Moreover, to ensure a 2-year follow-up, patients had to have undergone surgery by 2019 at the latest. Other exclusion criteria were age <16 years (as the treatment of patients with open growth zones varies); concomitant ligament injuries (injuries to the lateral collateral ligament, posterior cruciate ligament, and/or posterolateral ligament complex); fractures; tendon, vascular, or nerve injuries; other graft than hamstring, patellar, or quadriceps tendon autograft or missing graft choice; and missing surgeon code. Patients not completing the KOOS before the 2-year follow-up and patients undergoing subsequent knee surgery (revision or contralateral ACLR) within 2 years were excluded in the analysis of subjective knee function. Patients who underwent revision ACLR within 2 years from the primary index ACLR were analyzed separately.

### Exposure

Surgeons and clinics were categorized according to (1) their total registry caseload volume before the index ACLR and (2) their annual volume the year that the ACLR was performed. A combination of both variables reflects the surgeon’s/clinic’s previous and current experience/routine. Surgeons and clinics were therefore categorized into 4 groups, respectively: low total caseload and annual volume (LCLV), low total caseload and high annual volume (LCHV), high total caseload and low annual volume (HCLV), and finally, high total caseload and annual volume (HCHV). We used a cutoff for total registry caseload volume of ≥50 operations and a cutoff for annual volume of ≥29 ACLRs/year to define high-volume surgeons, according to a previous study based on the same population.^28^ High-volume clinics were defined as clinics with a total registry caseload of ≥500 previous surgeries (arbitrarily chosen cutoff) and clinics with an annual volume of ≥56 ACLRs/year (according to a previous study from the SNKLR).^28^

### Outcome Measures

The primary outcome of interest was the KOOS. The KOOS evaluates patients’ perception of knee function and knee-related problems on 5 subscales (Pain, Symptoms, Activities of Daily Living [ADL], Sports and Recreation [Sports/Rec], and Knee-Related Quality of Life [QoL]).^3,30^ We analyzed KOOS subscales preoperatively and 2 years postoperatively, with a main focus on the Sports/Rec and QoL subscales since they have the highest content validity and the greatest room for improvement among patients with ACL injuries.^3,14^ Analyses were also made using the KOOS_4_ (mean score of the KOOS Pain, Symptoms, Sports/Rec, and QoL subscales), thus excluding the subscale ADL, which has a low relevance and high ceiling effect in this study population.^3,7^ Finally, and most importantly, threshold values of MIC, PASS, and TF were applied to determine if the KOOS results were of clinically meaningful importance (Table 1).^13,14,29^

**Table 1:** KOOS Thresholds^*a*^

	MIC	PASS	TF
KOOS subscale^*b*^			
Pain	2.5	89	57
Symptoms	−1.2	83	56
ADL	2.4	95	71
Sports/Rec	12.1	72	28
QoL	18.3	73	28
KOOS_4_	9	79	42

a ADL, Activities of Daily Living; KOOS, Knee injury and Osteoarthritis Outcome Score; KOOS_4_, mean score of the KOOS Pain, Symptoms, Sports/Rec, and QoL subscales; MIC, minimal important change; PASS, Patient Acceptable Symptom State; QoL, Quality of Life; Sports/Rec, Sports and Recreation; TF, treatment failure.

b The range of each KOOS subscale is 0 to 100, where 0 = worst and 100 = best.

The secondary outcome was revision ACLR within 2 years from primary ACLR, and tertiary outcomes were time from injury to surgery, outpatient surgery, operating time, perioperative complications (as deemed relevant by the surgeon, such as graft contamination, technical failures, and equipment-related complications), and use of antibiotics and thromboprophylaxis.

### Statistical Analyses

SPSS Statistics Version 27 (IBM Corp) was used to conduct all analyses. Descriptive statistics were used to summarize all the variables. Median (interquartile range [IQR]) was reported for nonnormally distributed continuous variables, and the Kruskal-Wallis test was used for between-group comparisons. Counts and percentages were presented for categorical variables, and the chi-square test was used for between-group comparisons. Significant between-group differences were further evaluated with pairwise group comparisons using the Mann-Whitney *U* test (continuous variables) and the chi-square or Fisher exact test (categorical variables). A post hoc correction was conducted for pairwise comparisons (Bonferroni-adjusted *P* values).

An adjusted multivariable logistic regression analysis was used to examine if the patients’ odds of reaching MIC, PASS, or TF threshold values of the KOOS subscales and KOOS_4_ were influenced by surgeon and/or clinic volume, presented as the odds ratio with 95% CI. Age at surgery and patient sex were entered first, and then surgeon groups, clinic groups, and other potential confounding factors (body mass index [BMI], activity at time of injury, meniscal injury, chondral injury, MCL injury, time from injury to surgery, operating time, perioperative complications, ACL graft, and preoperative KOOS) were introduced in a forward stepwise method and retained in the model if the *P* value was <.10.

The occurrence of revision ACLR within 2 years from primary ACLR was examined for the surgeon and clinic groups, and an adjusted Cox regression analysis was conducted to determine the hazard ratio (HR) with 95% CI. The model included the same variables as were used in the logistic regression analyses. Statistical significance was set at a *P* value <.05 (2-tailed) in all analyses.

## Results

**Figure 1.**
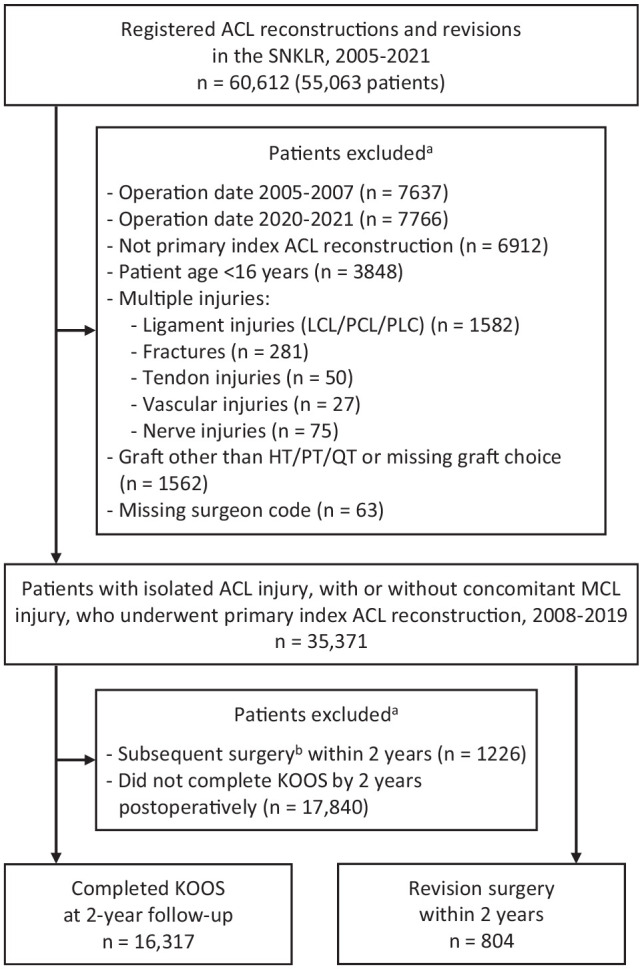
Flowchart of patient selection. ^a^Patients may appear in several groups. ^b^Subsequent surgery = revision or contralateral anterior cruciate ligament (ACL) reconstruction. HT, hamstring tendon; LCL, lateral collateral ligament; MCL, medial collateral ligament; PCL, posterior cruciate ligament; PLC, posterolateral complex; PT, patellar tendon; SNKLR, Swedish National Knee Ligament Registry; QT, quadriceps tendon.

There were 35,371 patients, 16,317 of whom completed the 2-year KOOS without having a subsequent surgery during the follow-up period (Figure 1). They underwent surgery by 278 surgeons at 91 clinics.

Patient characteristics are presented in Table 2, and all pairwise group comparisons are shown in Supplemental Material 1 (available separately). Surgeons and clinics with HCHV performed more outpatient surgeries, operated on their patients earlier (time from injury to surgery), and had a decreased operating time (all *P* < .001). The rates of perioperative complications and use of thromboprophylaxis and nonrecommended perioperative antibiotics were also lower among patients operated on by HCHV surgeons (all *P* < .001). Similar trends were seen for HCHV clinics compared with LCLV clinics (all *P* < .001).

**Table 2:** Baseline Patient Characteristics by Surgeon Group and Clinic Group^*a*^

Characteristic	By Surgeon Group^*b*^				
	LCLV (n = 1850)	LCHV (n = 575)	HCLV (n = 3466)	HCHV (n = 10,426)	*P*
Female sex	896 (48.4)	271 (47.1)	1664 (48.0)	5247 (50.3)	.045
Age at surgery, y	26 (20-37)	26 (20-37)	26 (20-38)	27 (20-39)	**<.001**
BMI, kg/m^2*c*^	24.7 (22.8-26.8)	24.1 (22.4-26.5)	24.4 (22.5-26.3)	24.2 (22.4-26.3)	**.001**
Activity at time of injury^*c*^					
Pivoting contact sport^*d*^	1072 (57.9)	315 (54.9)	1991 (57.4)	5569 (53.5)	**<.001**
Associated injury					
Meniscal	805 (43.5)	239 (41.6)	1514 (43.7)	4705 (45.1)	.149
Chondral	550 (29.7)	95 (16.5)	1008 (29.1)	2963 (28.4)	**<.001**
MCL	66 (3.6)	24 (4.2)	139 (4.0)	476 (4.6)	.180
Time to surgery, mo^*c*^	9.0 (5.4-17.3)	7.5 (4.1-14.1)	8.3 (4.5-17.0)	7.3 (4.0-14.5)	**<.001**
Outpatient surgery	1487 (80.4)	485 (84.3)	2925 (84.4)	9076 (87.1)	**<.001**
Operating time, min^*c*^	90 (74-110)	80 (64-100)	70 (60-90)	64 (50-80)	**<.001**
Perioperative complications	66 (3.6)	11 (1.9)	81 (2.3)	129 (1.2)	**<.001**
Perioperative antibiotics^*c*^					**<.001**
Cloxacillin	1718 (93.7)	541 (95.2)	3294 (96.0)	9944 (96.2)	
Clindamycin	35 (1.9)	22 (3.9)	91 (2.7)	347 (3.4)	
Other^*e*^	81 (4.4)	5 (0.9)	48 (1.4)	46 (0.4)	
Thromboprophylaxis	914 (49.4)	122 (21.2)	1273 (36.7)	2867 (27.5)	**<.001**
Graft choice					**<.001**
HT	1756 (94.9)	566 (98.4)	3297 (95.1)	9739 (93.4)	
PT	91 (4.9)	6 (1.0)	150 (4.3)	405 (3.9)	
QT	3 (0.2)	3 (0.5)	19 (0.5)	282 (2.7)	
	By Clinic Group^*f*^				
	LCLV (n = 4095)	LCHV (n = 3730)	HCLV (n = 453)	HCHV (n = 8039)	*P*
Female sex	2006 (49.0)	1810 (48.5)	223 (49.2)	4039 (50.2)	.304
Age at surgery, y	26.0 (20.0-38.0)	26.0 (20.0-36.0)	25.0 (19.0-36.0)	28.0 (21.0-39.0)	**<.001**
BMI, kg/m^2*c*^	24.5 (22.7-26.6)	24.3 (22.5-26.6)	24.5 (22.2-26.4)	24.2 (22.4-26.4)	**<.001**
Activity at time of injury^*c*^					
Pivoting contact sport^*d*^	2346 (57.3)	2189 (58.7)	271 (59.8)	4141 (51.6)	**<.001**
Associated injury					
Meniscal	1751 (42.8)	1578 (42.3)	207 (45.7)	3727 (46.4)	**<.001**
Chondral	1269 (31.0)	1093 (29.3)	109 (24.1)	2145 (26.7)	**<.001**
MCL	134 (3.3)	166 (4.5)	15 (3.3)	390 (4.9)	**<.001**
Time to surgery, mo^*c*^	8.8 (4.7-17.6)	7.8 (4.4-15.3)	8.3 (4.6-15.4)	7.1 (3.9-14.1)	**<.001**
Outpatient surgery	3258 (79.6)	3197 (85.7)	386 (85.2)	7132 (88.7)	**<.001**
Operating time, min^*c*^	76.0 (60.0-95.0)	65.0 (52.0-85.0)	70.0 (55.0-100.0)	65.0 (54.0-81.0)	**<.001**
Perioperative complications	109 (2.7)	86 (2.3)	14 (3.1)	78 (1.0)	**<.001**
Perioperative antibiotics^*c*^					**<.001**
Cloxacillin	3806 (93.7)	3464 (93.8)	438 (98.0)	7789 (97.7)	
Clindamycin	180 (4.4)	144 (3.9)	9 (2.0)	162 (2.0)	
Other^*e*^	78 (1.9)	83 (2.2)	0 (0.0)	19 (0.2)	
Thromboprophylaxis	1989 (48.6)	1260 (33.8)	157 (34.7)	1770 (22.0)	**<.001**
Graft choice					**<.001**
HT	3859 (94.2)	3571 (95.7)	426 (94.0)	7502 (93.3)	
PT	163 (4.0)	95 (2.5)	24 (5.3)	370 (4.6)	
QT	73 (1.8)	64 (1.7)	3 (0.7)	167 (2.1)	

a Data are reported as n (%) or median (IQR). Boldface *P* values indicate a statistically significant difference among groups (*P* < .05). BMI, body mass index; HCHV, high caseload and high volume; HCLV, high caseload and low volume; HT, hamstring tendon, LCHV, low caseload and high volume; LCLV, low caseload and low volume; MCL, medial collateral ligament; PT, patellar tendon; QT, quadriceps tendon.

b Number of surgeons: LCLV: 206; LCHV: 57; HCLV: 147; HCHV: 114. Each surgeon may appear in 1 or several groups.

c Missing patient values: BMI = 4773; activity at time of injury = 9; time to surgery = 1217; operating time = 765; perioperative antibiotics = 145.

d Pivoting sports = soccer, floorball, handball, hockey, American football/rugby, basketball.

e Other nonrecommended antibiotics, for example, cefuroxim.

f Number of clinics: LCLV: 78; LCHV: 45; HCLV: 14; HCHV: 26. Each clinic may appear in 1 or several groups.

### KOOS Results

**Figure 2.**
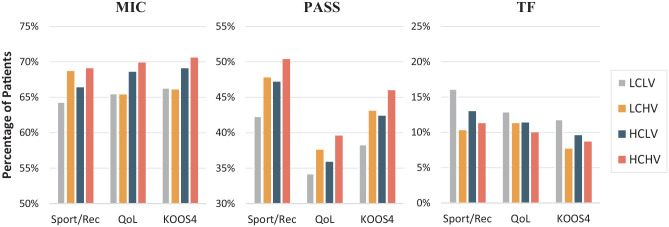
Percentage of patients meeting criteria of minimal important change (MIC), Patient Acceptable Symptom State (PASS), and treatment failure (TF) of the Knee injury and Osteoarthritis Outcome Score (KOOS) Sports and Recreation (Sports/Rec) and Quality of Life (QoL) subscales and KOOS_4_ (mean score of the KOOS Pain, Symptoms, Sports/Rec, and QoL subscales) categorized by surgeon experience when primary anterior cruciate ligament reconstruction was conducted. Range 0 to 100, worst to best. Note that the y-axis starts at other values than zero in MIC and PASS. HCHV, high caseload and high volume; HCLV, high caseload and low volume; LCHV, low caseload and high volume; LCLV, low caseload and low volume.

The results of all KOOS subscales and KOOS_4_ (preoperative and 2-year postoperative score and median difference of improvement and MIC, PASS, and TF rates with pairwise group comparisons) are presented in Supplemental Material 2 (available separately). Of the included patients, 12,139 (74.4%) had completed preoperative KOOS. The proportion of 2-year KOOS responders by surgeon and clinic groups is presented in Supplemental Material 3 (available separately). The median differences of improvement in KOOS Sports/Rec and KOOS QoL, as well as in KOOS_4_, were better if the primary ACLR was conducted by HCHV surgeons compared with surgeons in the LCLV group: Sports/Rec: 30.0 (IQR, 5.0-50.0) versus 25.0 (IQR, 5.0-40.0) (*P* = .012); QoL: 31.3 (IQR, 12.5-43.8) versus 25.0 (IQR, 6.3-43.3) (*P* = .002); and KOOS_4_: 19.5 (IQR, 6.3-32.4) versus 17.2 (IQR, 4.2-30.7) (*P* = .010). There were no significant differences of improvement in KOOS Sports/Rec, KOOS QoL, or KOOS_4_ among the clinic groups. A higher percentage of patients operated on by HCHV surgeons fulfilled the criteria of MIC and PASS in KOOS Sports/Rec, KOOS QoL, and KOOS_4_, whereas a higher proportion of patients operated on by LCLV surgeons were within the range of TF (HCHV surgeons vs LCLV surgeons; all *P* < .02) (Figure 2).

Comparisons of clinic groups showed no significant differences in the proportion of patients who achieved the MIC for KOOS Sports/Rec, KOOS QoL, or KOOS_4_. However, patients operated on at HCHV clinics met the PASS on all 3 KOOS measures more often than those operated on at LCLV clinics: KOOS Sports/Rec: 51.3% versus 44.8% (*P* < .001); KOOS QoL: 39.1% versus 35.1% (*P* < .001); and KOOS_4_: 46.8% versus 40.6% (*P* < .001). The proportion of patients having a TF on the measures was lower if operated on at HCHV clinics compared with LCLV clinics: KOOS Sports/Rec: 11.0% versus 13.9% (*P* < .001); KOOS QoL: 10.0% vs 12.1% (*P* = .005); and KOOS_4_: 8.3% versus 10.7% (*P* < .001).

In the adjusted multivariable logistic regression analyses, surgeon experience influenced the odds of achieving MIC (Table 3) and PASS (Table 4) on the KOOS Sports/Rec and KOOS_4_ at the 2-year follow-up, whereas it did not influence PASS achievement on the KOOS QoL or the TF on the KOOS Sports/Rec, KOOS QoL, or KOOS_4_. Clinic volume had no influence on the odds of achieving MIC, PASS, or TF on any of the 3 KOOS measures. The adjusted multivariable logistic regression analyses not presented within the paper are shown in Supplemental Material 4 (available separately).

**Table 3:** Results of Adjusted Logistic Regression Analyses of Factors Influencing the MIC on the KOOS Sports/Rec, KOOS QoL, and KOOS_4_^*a*^

Variable	KOOS Sports/Rec (n = 8307)		KOOS QoL (n = 8314)		KOOS_4_ (n = 8295)	
	OR (95% CI)	*P*	OR (95% CI)	*P*	OR (95% CI)	*P*
Age	1.016 (1.010-1.021)	**<.001**	1.035 (1.030-1.041)	**<.001**	1.031 (1.026-1.036)	**<.001**
Female sex	0.742 (0.665-0.828)	**<.001**	0.926 (0.836-1.027)	.114	0.804 (0.724-0.893)	**<.001**
BMI	0.953 (0.937-0.970)	**<.001**	—		0.960 (0.944-0.976)	**<.001**
Chondral injury	0.749 (0.662-0.847)	**<.001**	0.797 (0.708-0.897)	**<.001**	0.811 (0.719-0.916)	**<.001**
Time to surgery	—		—		0.998 (0.997-0.999)	**.014**
ACL graft						
PT	0.641 (0.493-0.834)	**<.001**	0.694 (0.542-0.889)	**.004**	0.666 (0.518-0.856)	**.001**
QT	0.812 (0.549-1.201)		0.877 (0.606-1.268)	.485	0.791 (0.543-1.154)	.224
HT	Ref		Ref		Ref	
Preop KOOS						
Sports/Rec	0.962 (0.960-0.964)	**<.001**	—		—	
QoL	—		0.966 (0.963-0.969)	**<.001**	—	
KOOS_4_	—		—		0.957 (0.954-0.960)	**<.001**
Surgeon experience						
LCLV	0.705 (0.588-0.845)	**<.001**	0.774 (0.653-0.919)	**.003**	0.740 (0.621-0.881)	**<.001**
LCHV	0.969 (0.714-1.316)	.842	0.806 (0.608-1.069)	.135	0.763 (0.574-1.016)	.064
HCLV	0.826 (0.725-0.942)	**.004**	0.914 (0.806-1.037)	.163	0.867 (0.763-0.985)	**.029**
HCHV	Ref		Ref		Ref	

a Boldface *P* values indicate statistical significance (*P* < .05). ACL, anterior cruciate ligament; BMI, body mass index; HCHV, high caseload and high volume; HCLV, high caseload and low volume; HT, hamstring tendon; KOOS, Knee injury and Osteoarthritis Outcome Score; KOOS_4_, mean score of the KOOS Pain, Symptoms, Sports/Rec, and QoL subscales; LCLV, low caseload and low volume; LCHV, low caseload and high volume; MIC, minimal important change; PASS, Patient Acceptable Symptom State; Preop, preoperative; PT, patellar tendon; QoL, Quality of Life; QT, quadriceps tendon; Sports/Rec, Sports and Recreation.

**Table 4:** Results of Adjusted Logistic Regression Analyses of Factors Influencing the PASS on the KOOS Sports/Rec and KOOS_4_^*a*^

Variable	KOOS Sports/Rec (n = 8307)		KOOS_4_ (n = 8295)	
	OR (95% CI)	*P*	OR (95% CI)	*P*
Age	1.020 (1.015-1.024)	**<.001**	1.029 (1.024-1.033)	**<.001**
Female	0.749 (0.682-0.824)	**<.001**	0.810 (0.736-0.891)	**<.001**
BMI	0.957 (0.943-0.972)	**<.001**	0.965 (0.951-0.980)	**<.001**
Chondral injury	0.764 (0.685-0.851)	**<.001**	0.796 (0.714-0.888)	**<.001**
Time to surgery	0.998 (0.997-0.999)	**.006**	—	
ACL graft				
PT	0.631 (0.496-0.801)	**<.001**	0.713 (0.559-0.908)	**.006**
QT	0.718 (0.508-1.015)	.060	0.827 (0.582-1.175)	.289
HT	Ref		Ref	
Periop complications	0.650 (0.441-0.960)	**.030**	0.655 (0.439-0.979)	**.039**
Preop KOOS Sports/Rec	1.023 (1.022-1.025)	**<.001**	—	
Preop KOOS_4_	—		1.041 (1.038-1.044)	**<.001**
Surgeon experience				
LCLV	0.731 (0.621-0.861)	**<.001**	0.713 (0.604-0.841)	**<.001**
LCHV	0.911 (0.697-1.190)	.493	0.873 (0.666-1.144)	.325
HCLV	0.942 (0.840-1.058)	.314	0.937 (0.835-1.053)	.276
HCHV	Ref		Ref	

a Boldface *P* values indicate statistical significance (*P* < .05). ACL, anterior cruciate ligament, BMI, body mass index; HCHV, high caseload and high volume; HCLV, high caseload and low volume; HT, hamstring tendon; KOOS, Knee injury and Osteoarthritis Outcome Score; KOOS_4_, mean score of the KOOS Pain, Symptoms, Sports/Rec, and QoL subscales; LCLV, low caseload and low volume; LCHV, low caseload and high volume; MIC, minimal important change; PASS, Patient Acceptable Symptom State; Periop, perioperative; Preop, preoperative; PT, patellar tendon; QoL, Quality of Life; QT, quadriceps tendon; Sports/Rec, Sports and Recreation.

### Revision ACLR

There were 804 patients (2.3%) who underwent revision ACLR within 2 years from primary ACLR (Figure 1). No significant differences in crude revision rates were seen in the surgeon groups, but ACLR revision rates were higher among patients who underwent primary ACLR at HCHV clinics (2.5%) than at LCLV clinics (1.7%) (*P* < .001). However, in the adjusted Cox regression analysis (389 patients with revision ACLR within 2 years and 14,206 patients without), surgeon and clinic groups had no influence on the subsequent revision rate. Instead, we found that patient age (HR, 0.94; 95% CI, 0.93-0.96), time from injury to surgery (HR, 0.98; 95% CI, 0.97-0.99), injury during pivoting contact sports (HR, 1.31; 95% CI, 1.02-1.67), and preoperative KOOS_4_ (HR, 0.993; 95% CI, 0.987-0.999) significantly influenced the ACLR revision rate.

## Discussion

The most important finding of this study was that patients operated on by high-volume surgeons had increased odds of achieving a clinically relevant improvement (MIC) in KOOS Sports/Rec, KOOS QoL, and KOOS_4_, and reaching levels of satisfaction (PASS) in KOOS Sports/Rec and KOOS_4_, at the 2-year follow-up compared with patients operated on by low-volume surgeons. Surgical volume had no influence on subsequent ACLR revision rates when adjusting for demographic differences.

### Subjective Knee Function

Surgeon volume was found to influence subjective knee function after primary ACLR. Increased odds of achieving a clinically relevant improvement in KOOS Sports/Rec, KOOS QoL, and KOOS_4_ and reaching levels of satisfaction in KOOS Sports/Rec and KOOS_4_ were seen among patients operated on by HCHV surgeons compared with LCLV surgeons, at the 2-year follow-up. Even if there were significant differences in the proportion of patients deeming the outcome as a TF between HCHV and LCLV surgeons, factors other than surgeon volume (such as age, sex, BMI, chondral injury, and preoperative KOOS) were found to influence TF in KOOS Sports/Rec, KOOS QoL, and KOOS_4_. Regarding the clinics, a significantly higher proportion of patients fulfilled PASS, and a lower proportion reached TF if operated on at HCHV clinics compared with LCLV clinics. However, clinic volume had no influence on subjective knee function in the multivariable logistic regression analyses. Thus, our first hypothesis was only partially supported, as patients operated on by high-volume surgeons had significantly improved subjective outcomes, while clinic volume did not appear to have any impact.

The relationship between surgical volume and patient-reported outcomes is underinvestigated in the orthopaedic literature. Studies suggest that higher volume is linked to better patient-reported outcomes for procedures such as total knee arthroplasty and lumbar spine fusion, but not for total hip arthroplasty.^11,17,24^ To the best of our knowledge, this is the first study to investigate the relationship between surgeon volume and patient-reported outcomes in primary ACLR. Clinic volume was assessed in a recently published study from the Norwegian Knee Ligament Registry, where the authors found an increased improvement in 5-year KOOS QoL and a decreased rate of TFs (defined as QoL <44) among patients operated on at clinics with higher ACLR volume.^23^ The clinical relevance of these findings is, however, unclear since potential confounders were not controlled for. Previously, factors such as younger age, male sex, hamstring tendon autograft, absence of concomitant injuries, and decreased time from injury to surgery have been associated with improved patient-reported outcomes after primary ACLR.^1,8,26^ These findings are in accordance with the results of the present study. In addition to surgeon volume, we also identified BMI, perioperative complications, and preoperative KOOS as variables influencing subjective improvement and satisfaction, suggesting that these factors should be considered when model fitting in the setting of ACLR and when managing patient expectations preoperatively.

### Revision ACLR

The ACLR revision rate found in this study (2.3%) is in line with previous studies with a follow-up of ≤2 years (1.7%-2.5%).^5,20,22,32^ Interestingly, a slightly higher crude percentage of revision ACLR was found among patients operated on at HCHV clinics compared with LCLV clinics (2.5% vs 1.7%; *P* < .001). Surgical volume, however, had no influence on the revision rate when controlling for potential confounders. Instead, we found that younger age, decreased time from injury to surgery, injury during pivoting contact sports (soccer, floorball, handball, hockey, American football, rugby, and basketball), and lower preoperative KOOS_4_ significantly increased the HR of ACLR revision. Several of these factors have repeatedly been associated with an increased revision rate.^5,26,32,33^ Consequently, the increased crude revision rate seen among HCHV clinics is possibly explained by other factors, such as patient characteristics or that HCHV surgeons/clinics are more prone to proceed with revision surgery if needed, rather than technical failures. Nevertheless, our second hypothesis was not supported, as surgeon and clinic volume did not have any influence on ACLR revision rates when adjusting for confounders.

Similar to our findings, a study from the Norwegian Knee Ligament Registry found increased crude revision rates in patients operated on at high-volume clinics.^23^ However, in contrast to our results, the Norwegian study reported an adjusted HR of 1.6 for undergoing subsequent revision surgery when ACLR was performed at high-volume clinics (>100 ACLRs/year) compared with low-volume clinics (<13 ACLRs/year).^23^ These contradictory findings could be attributed to differences in patient age, follow-up times, cutoffs regarding clinic volume, and confounders used in the Cox regression models. However, another registry study from New Zealand found no association between surgical volume and subsequent revision surgery,^33^ while a report from the Danish Knee Ligament Registry showed that ACLR with quadriceps tendon autograft was associated with an increased revision rate if conducted by low-volume clinics (total caseload of <100 ACLRs).^19^ Finally, an increased risk of subsequent ACLR among low-volume clinics (<125 ACLRs/year) was reported in a registry study from the United States, but the distinction between revision and contralateral ACLR was not conducted, making comparisons to our and other studies difficult.^21^

### Management of Primary ACLR

Interestingly, a decreased time from injury to surgery, shorter operating time, fewer perioperative complications, and a higher proportion of outpatient surgery were seen in patients operated on by HCHV surgeons or at HCHV clinics. These findings extend previous literature that high-volume surgeons/clinics provide care more efficiently.^4,18,31^

High-volume surgeons/clinics prescribed more perioperative cloxacillin (which is the preferred first-line infection prophylaxis in Sweden in the absence of a type 1 penicillin allergy) than low-volume surgeons/clinics, possibly indicating greater compliance with national and/or regional guidelines among high-volume providers. Another interesting finding was the discrepancy found in the use of postoperative thromboprophylaxis, where surgeons/clinics with a low annual surgical volume prescribed thromboprophylaxis more frequently. A prior study from the SNKLR reported that venous thromboembolism followed ACLR in 0.4% of cases,^15^ and a survey among Swedish ACL surgeons found that thromboprophylaxis was only prescribed to patients with known risk factors for venous thromboembolism.^6^ Thus, the observed differences in this study might be explained by patient-related factors (such as prior thrombosis, family history of thrombosis, use of contraceptives, smoking, and being overweight) or, more likely, by clinic routines, use of postoperative knee bracing, or prolonged operating time.^6^ Arthroscopic procedures of the knee taking >90 minutes, which was the median operating time for LCLV surgeons, are considered a risk factor for pulmonary embolism.^10^

### Strengths and Limitations

The main strength of this study was the large sample size from a nationwide registry, making the results more generalizable to the population undergoing primary ACLR. Although the underlying cause of the reported results is multifactorial, and surgical volume cannot be considered the sole determinant of improved outcomes, our study provides valuable insights by establishing a clear association between surgeon volume and patient-reported subjective knee function. However, the interpretation regarding the influence of surgical volume on subjective knee function might be somewhat limited because of nonresponse and missing data bias. In an attempt to adjust for confounders, such as preoperative KOOS, BMI, and time from injury to surgery, nearly half of the patients were excluded from the multivariable logistic regression analyses. Approximately 26% of patients did not complete the preoperative KOOS. While the proportion of preoperative nonresponders was higher within the low-volume categories (LCLV surgeons: 36.0%; LCLV clinics: 39.8%), more than half of the patients not completing preoperative KOOS underwent surgery by HCHV surgeons and approximately one-third underwent surgery by HCHV clinics. However, high-volume surgeons and clinics likely have more appropriate methods of enrolling patients into the SNKLR before surgery. A previous study from the SNKLR identified a decreased response rate among younger patients, male patients, and possibly patients with lower socioeconomic status, although they reported no clinically relevant differences in KOOS subscales between responders and nonresponders.^27^ Interestingly, we found no differences in response rates among the surgeon groups at the 2-year follow-up, suggesting that loss to follow-up did not influence our results.

Furthermore, the use of KOOS and other patient-reported outcome measures has been questioned lately because of inconsistent content validity.^9^ While this might be true for the KOOS subscale ADL in a young population undergoing ACLR, the Sports/Rec and QoL subscales have been found to have an adequate content validity in this setting.^3,34^ Next, the threshold values of MIC, PASS, and TF vary across the literature, and there is no established consensus. However, we applied thresholds derived from studies with similar patient populations.^13,14,29^ Despite the incorporation of MIC, PASS, and TF, it is important to acknowledge that the clinical relevance of our results might have been biased by the patients’ overall treatment experience, and not solely by the ACLR itself. It is plausible that patients who underwent surgery by high-volume surgeons at clinics with superior multidisciplinary approaches and access to structured rehabilitation may have had more positive experiences, leading to improved subjective outcomes.

In addition, the nature of registry data prohibited us from controlling for factors that are not measured or reported but that might be of importance, such as postoperative rehabilitation protocol, presence of graft failure among patients not proceeding with revision ACLR, and indication for revision ACLR. The lack of patient activity levels is another limitation to consider, since it might have affected subjective outcomes as well as revision rates. However, even if we were able to adjust for activity levels, patient demands and expectations are complex and difficult to account for and could confound the results in several ways. If we speculate, highly active patients with great demands on their knee function might be more fit, motivated, and prone to return to preinjury activity levels, thus scoring higher on both pre- and postoperative KOOS compared with patients with lower activity levels and non–knee-demanding labor. On the contrary, highly active patients might have overly high expectations, making the KOOS lower, whereas less active patients might be more than satisfied that they can return to their ordinary work and therefore score higher. Finally, there is no accepted definition of high- and low-volume surgeons or clinics in the setting of ACLR; thus, the generalizability of our results may vary depending on the surgical volumes in each country.

## Conclusion

Patients having primary ACLR by high-volume surgeons experienced increased improvement and satisfaction regarding subjective knee function compared with patients operated on by low-volume surgeons. Other factors, rather than surgeon and clinic volume, were found to influence subsequent revision rates. In all, patients might benefit if operated on by high-volume providers.

## Supplemental Material

sj-pdf-1-ojs-10.1177_23259671241233695 – Supplemental material for Influence of Surgeon Experience and Clinic Volume on Subjective Knee Function and Revision Rates in Primary ACL Reconstruction: A Study from the Swedish National Knee Ligament Registry Supplemental material, sj-pdf-1-ojs-10.1177_23259671241233695 for Influence of Surgeon Experience and Clinic Volume on Subjective Knee Function and Revision Rates in Primary ACL Reconstruction: A Study from the Swedish National Knee Ligament Registry by Dzan Rizvanovic, Markus Waldén, Magnus Forssblad and Anders Stålman in Orthopaedic Journal of Sports Medicine

sj-pdf-2-ojs-10.1177_23259671241233695 – Supplemental material for Influence of Surgeon Experience and Clinic Volume on Subjective Knee Function and Revision Rates in Primary ACL Reconstruction: A Study from the Swedish National Knee Ligament Registry Supplemental material, sj-pdf-2-ojs-10.1177_23259671241233695 for Influence of Surgeon Experience and Clinic Volume on Subjective Knee Function and Revision Rates in Primary ACL Reconstruction: A Study from the Swedish National Knee Ligament Registry by Dzan Rizvanovic, Markus Waldén, Magnus Forssblad and Anders Stålman in Orthopaedic Journal of Sports Medicine

sj-pdf-3-ojs-10.1177_23259671241233695 – Supplemental material for Influence of Surgeon Experience and Clinic Volume on Subjective Knee Function and Revision Rates in Primary ACL Reconstruction: A Study from the Swedish National Knee Ligament Registry Supplemental material, sj-pdf-3-ojs-10.1177_23259671241233695 for Influence of Surgeon Experience and Clinic Volume on Subjective Knee Function and Revision Rates in Primary ACL Reconstruction: A Study from the Swedish National Knee Ligament Registry by Dzan Rizvanovic, Markus Waldén, Magnus Forssblad and Anders Stålman in Orthopaedic Journal of Sports Medicine

sj-pdf-4-ojs-10.1177_23259671241233695 – Supplemental material for Influence of Surgeon Experience and Clinic Volume on Subjective Knee Function and Revision Rates in Primary ACL Reconstruction: A Study from the Swedish National Knee Ligament Registry Supplemental material, sj-pdf-4-ojs-10.1177_23259671241233695 for Influence of Surgeon Experience and Clinic Volume on Subjective Knee Function and Revision Rates in Primary ACL Reconstruction: A Study from the Swedish National Knee Ligament Registry by Dzan Rizvanovic, Markus Waldén, Magnus Forssblad and Anders Stålman in Orthopaedic Journal of Sports Medicine
