# Supplementary material for: Influence of Surgeon Experience and Clinic Volume on Subjective Knee Function and Revision Rates in Primary ACL Reconstruction: A Study from the Swedish National Knee Ligament Registry
Source: Orthop J Sports Med. 2024 Mar 11;12(3):23259671241233695. doi: 10.1177/23259671241233695 (PMC10929050; doi:10.1177/23259671241233695)
Supplement: sj-pdf-1-ojs-10.1177_23259671241233695 – Supplemental material for Influence of Surgeon Experience and Clinic Volume on Subjective Knee Function and Revision Rates in Primary ACL Reconstruction: A Study from the Swedish National Knee Ligament Registry [file sj-pdf-1-ojs-10.1177_23259671241233695.pdf]

Supplemental Material 1: Baseline data with group comparisons

Baseline data presented by surgeon groups[a]

	LCLV	LCHV	HCLV	HCHV		Bonferroni-adjusted *P* values					
	(n = 1850)	(n = 575)	(n = 3466)	(n = 10,426)	*P* value	LCLV vs LCHV	LCLV vs HCLV	LCLV vs HCHV	LCHV vs HCLV	LCHV vs HCHV	HCLV vs HCHV
Females	896 (48.4)	271 (47.1)	1,664 (48.0)	5,247 (50.3)	0.045	>0.999	>0.999	0.820	>0.999	0.872	0.112
Age at surgery, years	26 (20-37)	26 (20-37)	26 (20-38)	27 (20-39)	<0.001	>0.999	0.565	<0.001	>0.999	0.499	0.040
BMI[b]	24.7 (22.8-26.8)	24.1 (22.4-26.5)	24.4 (22.5-26.3)	24.2 (22.4-26.3)	0.001	0.147	0.028	<0.001	>0.999	>0.999	>0.999
Activity at time of injury[b]											
Pivoting contact sport[c]	1,072 (57.9)	315 (54.9)	1,991 (57.4)	5,569 (53.5)	<0.001	>0.999	>0.999	0.002	>0.999	>0.999	<0.001
Associated injury											
Meniscal injury	805 (43.5)	239 (41.6)	1,514 (43.7)	4,705 (45.1)	0.149						
Chondral injury	550 (29.7)	95 (16.5)	1,008 (29.1)	2,963 (28.4)	<0.001	<0.001	>0.999	>0.999	<0.001	<0.001	>0.999
MCL injury	66 (3.6)	24 (4.2)	139 (4.0)	476 (4.6)	0.180						
Time to surgery, months[b]	9.0 (5.4-17.3)	7.5 (4.1-14.1)	8.3 (4.5-17.0)	7.3 (4.0-14.5)	<0.001	<0.001	0.004	<0.001	0.078	>0.999	<0.001
Outpatient surgery	1,487 (80.4)	485 (84.3)	2,925 (84.4)	9,076 (87.1)	<0.001	0.223	0.001	<0.001	>0.999	0.379	<0.001
Operating time, min[b]	90 (74-110)	80 (64-100)	70 (60-90)	64 (50-80)	<0.001	<0.001	<0.001	<0.001	<0.001	<0.001	<0.001
Perioperative complications	66 (3.6)	11 (1.9)	81 (2.3)	129 (1.2)	<0.001	0.334	0.065	<0.001	>0.999	>0.999	<0.001
Perioperative antibiotics[b]					<0.001	<0.001	<0.001	<0.001	0.996	>0.999	<0.001
Cloxacillin	1,718 (93.7)	541 (95.2)	3,294 (96.0)	9,944 (96.2)							
Clindamycin	35 (1.9)	22 (3.9)	91 (2.7)	347 (3.4)							
Other[d]	81 (4.4)	5 (0.9)	48 (1.4)	46 (0.4)							
Thromboprophylaxis, yes	914 (49.4)	122 (21.2)	1273 (36.7)	2,867 (27.5)	<0.001	<0.001	<0.001	<0.001	<0.001	0.006	<0.001
Graft choice					<0.001	<0.001	0.429	<0.001	0.005	<0.001	<0.001
HT	1,756 (94.9)	566 (98.4)	3,297 (95.1)	9,739 (93.4)							
PT	91 (4.9)	6 (1.0)	150 (4.3)	405 (3.9)							
QT	3 (0.2)	3 (0.5)	19 (0.5)	282 (2.7)							

Data are reported as n (%) or median (25th-75th percentile). BMI, body mass index; HCHV, high caseload and high annual volume; HCLV, high caseload and low annual volume; HT, hamstring tendon, LCHV, low caseload and high annual volume; LCLV, low caseload and low annual volume; MCL, medial collateral ligament; min, minutes; PT, patellar tendon; QT, quadriceps tendon.

a. N surgeons – LCLV: 206, LCHV: 57, HCLV: 147, HCHV: 114. Each surgeon may appear in one or several groups.

b. Missing values: Activity at time of injury – 9 (LCLV: 1, HCHV: 8), BMI – 4,773 (LCLV: 684, LCHV: 189, HCLV: 1,116, HCHV: 2,784), Time to surgery – 1,217 (LCLV: 160, LCHV: 54, HCLV: 232, HCHV: 771), Operating time – 765 (LCLV: 64, LCHV: 33, HCLV: 152, HCHV: 516), Perioperative antibiotics – 145 (LCLV: 16, LCHV: 7, HCLV: 33, HCHV: 89).

c. Pivoting contact sport = soccer, floorball, handball, American football/rugby, basketball.

d. Other = Other non-recommended antibiotics, for example Cefuroxim.

Baseline data presented by clinic groups[a]

	LCLV (n = 4095)	LCHV (n = 3730)	HCLV (n = 453)	HCHV (n = 8039)	*P* value	Bonferroni-adjusted *P* values					
						LCLV vs LCHV	LCLV vs HCLV	LCLV vs HCHV	LCHV vs HCLV	LCHV vs HCHV	HCLV vs HCHV
Females	2,006 (49.0)	1,810 (48.5)	223 (49.2)	4,039 (50.2)	0.304						
Age at surgery, years	26.0 (20.0-38.0)	26.0 (20.0-36.0)	25.0 (19.0-36.0)	28.0 (21.0-39.0)	<0.001	0.499	0.804	<0.001	>0.999	<0.001	0.002
BMI[b]	24.5 (22.7-26.6)	24.3 (22.5-26.6)	24.5 (22.2-26.4)	24.2 (22.4-26.2)	<0.001	0.259	>0.999	<0.001	>0.999	0.296	>0.999
Activity at time of injury[b]											
Pivoting contact sport[c]	2,346 (57.3)	2,189 (58.7)	271 (59.8)	4,141 (51.6)	<0.001	>0.999	>0.999	<0.001	>0.999	<0.001	0.004
Associated injury											
Meniscal injury	1,751 (42.8)	1.578 (42.3)	207 (45.7)	3,727 (46.4)	<0.001	>0.999	>0.999	<0.001	>0.999	<0.001	>0.999
Chondral injury	1,269 (31.0)	1,093 (29.3)	109 (24.1)	2,145 (26.7)	<0.001	0.654	0.012	0.001	0.125	0.019	>0.999
MCL injury	134 (3.3)	166 (4.5)	15 (3.3)	390 (4.9)	<0.001	0.047	>0.999	<0.001	>0.999	>0.999	>0.999
Time to surgery, months[b]	8.8 (4.7-17.6)	7.8 (4.4-15.3)	8.3 (4.6-15.4)	7.1 (3.9-14.1)	<0.001	<0.001	>0.999	<0.001	>0.999	<0.001	0.046
Outpatient surgery	3,258 (79.6)	3,197 (85.7)	386 (85.2)	7.132 (88.7)	<0.001	<0.001	0.026	<0.001	>0.999	<0.001	0.167
Operating time, min[b]	76.0 (60.0-95-0)	65.0 (52.0-85.0)	70.0 (55.0-100.0)	65.0 (54.0-81.0)	<0.001	<0.001	>0.999	<0.001	<0.001	>0.999	<0.001
Perioperative complications	109 (2.7)	86 (2.3)	14 (3.1)	78 (1.0)	<0.001	>0.999	>0.999	<0.001	>0.999	<0.001	0.002
Perioperative antibiotics[b]					<0.001	>0.999	0.003	<0.001	0.004	<0.001	>0.999
Cloxacillin	3,806 (93.7)	3,464 (93.8)	438 (98.0)	7,789 (97.7)							
Clindamycin	180 (4.4)	144 (3.9)	9 (2.0)	162 (2.0)							
Other[d]	78 (1.9)	83 (2.2)	0 (0)	19 (0.2)							
Thromboprophylaxis, yes	1,989 (48.6)	1,260 (33.8)	157 (34.7)	1,770 (22.0)	<0.001	<0.001	<0.001	<0.001	>0.999	<0.001	<0.001
Graft choice					<0.001	0.011	0.548	0.886	0.007	<0.001	0.555
HT	3,859 (94.2)	3,571 (95.7)	426 (94.0)	7,502 (93.3)							
PT	163 (4.0)	95 (2.5)	24 (5.3)	370 (4.6)							
QT	73 (1.8)	64 (1.7)	3 (0.7)	167 (2.1)							

Data are reported as n (%) or median (25th-75th percentile). BMI, body mass index; HCHV, high caseload and high annual volume; HCLV, high caseload and low annual volume; HT, hamstring tendon, LCHV, low caseload and high annual volume; LCLV, low caseload and low annual volume; MCL, medial collateral ligament; min, minutes; PT, patellar tendon; QT, quadriceps tendon.

a. N surgeons – LCLV: 206, LCHV: 57, HCLV: 147, HCHV: 114. Each surgeon may appear in one or several groups.

b. Missing values: Activity at time of injury – 9 (LCLV: 1, HCHV: 8), BMI – 4,773 (LCLV: 684, LCHV: 189, HCLV: 1,116, HCHV: 2,784), Time to surgery – 1,217 (LCLV: 160, LCHV: 54, HCLV: 232, HCHV: 771), Operating time – 765 (LCLV: 64, LCHV: 33, HCLV: 152, HCHV: 516), Perioperative antibiotics – 145 (LCLV: 16, LCHV: 7, HCLV: 33, HCHV: 89).

c. Pivoting contact sport = soccer, floorball, handball, American football/rugby, basketball.

d. Other = Other non-recommended antibiotics, for example Cefuroxim.
