# Supplementary material for: Influence of Surgeon Experience and Clinic Volume on Subjective Knee Function and Revision Rates in Primary ACL Reconstruction: A Study from the Swedish National Knee Ligament Registry
Source: Orthop J Sports Med. 2024 Mar 11;12(3):23259671241233695. doi: 10.1177/23259671241233695 (PMC10929050; doi:10.1177/23259671241233695)
Supplement: sj-pdf-2-ojs-10.1177_23259671241233695 – Supplemental material for Influence of Surgeon Experience and Clinic Volume on Subjective Knee Function and Revision Rates in Primary ACL Reconstruction: A Study from the Swedish National Knee Ligament Registry [file sj-pdf-2-ojs-10.1177_23259671241233695.pdf]

Supplemental Material 2: KOOS data with group comparisons

KOOS data presented by surgeon groups

	Overall	LCLV	LCHV	HCLV	HCHV		Bonferroni-adjusted *P* values					
	(N = 16,317)	(n = 1850)	(n = 575)	(n = 3466)	(n = 10,426)	*P* value	LCLV vs LCHV	LCLV vs HCLV	LCLV vs HCHV	LCHV vs HCLV	LCHV vs HCHV	HCLV vs HCHV
Preoperative KOOS, yes (%)	12,139 (74.4)	1,184 (64.0)	387 (67.3)	2,480 (71.6)	8,088 (77.6)	<0.001	0.970	<0.001	<0.001	0.252	<0.001	<0.001
Pain	12.134	75.0 (61.1-86.1)	80.6 (66.7-88.9)	75.0 (61.1-86.1)	77.8 (63.9-88.9)	<0.001	0.004	>0.999	0.011	0.014	0.244	0.038
Symptoms	12.136	67.9 (53.6-82.1)	75.0 (57.1-85.7)	71.4 (57.1-82.1)	71.4 (57.1-85.7)	<0.001	0.017	>0.999	0.002	0.073	>0.999	0.008
ADL	12.134	86.8 (70.6-95.6)	91.2 (76.5-97.1)	88.2 (72.1-95.6)	88.2 (73.5-97.1)	<0.001	0.009	>0.999	0.010	0.034	0.507	0.032
Sport/Rec	12.116	35.0 (15.0-55.0)	40.0 (20.0-60.0)	35.0 (15.0-60.0)	35.0 (15.0-60.0)	0.033	0.370	>0.999	0.107	0.907	>0.999	0.381
QoL	12.128	31.3 (18.8-43.8)	31.3 (25.0-43.4)	31.3 (18.8-43.8)	31.3 (18.8-43.8)	0.010	0.213	>0.999	0.575	0.100	>0.999	0.053
KOOS4	12.109	52.5 (40.4-65.4)	55.8 (43.7-68.1)	53.4 (40.5-65.8)	54.6 (41.8-66.9)	<0.001	0.023	>0.999	0.013	0.059	0.993	0.019
Two-year KOOS												
Pain	16.312	86.1 (75.0-94.4)	88.9 (77.8-97.2)	88.9 (75.0-97.2)	88.9 (77.8-97.2)	<0.001	0.020	0.017	<0.001	>0.999	>0.999	<0.001
Symptoms	16.314	78.6 (64.3-89.3)	82.1 (67.9-92.9)	82.1 (64.3-92.9)	82.1 (67.9-92.9)	<0.001	0.291	0.011	<0.001	>0.999	0.391	<0.001
ADL	16.311	95.6 (86.8-100.0)	97.1 (89.7-100.0)	97.1 (88.2-100.0)	97.1 (86.7-100.0)	<0.001	0.005	<0.001	<0.001	>0.999	>0.999	0.017
Sport/Rec	16.301	70.0 (40.0-85.0)	70.0 (45.0-85.0)	70.0 (45.0-85.0)	75.0 (50.0-90.0)	<0.001	0.043	0.002	<0.001	>0.999	0.911	<0.001
QoL	16.302	62.5 (37.5-75.0)	62.5 (43.8-81.3)	62.5 (43.8-75.0)	62.5 (43.8-81.3)	<0.001	0.275	0.073	<0.001	>0.999	0.706	<0.001
KOOS4	16.285	72.8 (55.7-85.3)	75.2 (59.9-87.6)	75.0 (58.3-86.9)	77.0 (61.3-88.3)	<0.001	0.059	0.005	<0.001	>0.999	0.660	<0.001
KOOS difference												
Pain	12.130	8.3 (0.0-19.4)	8.3 (-2.8-19.4)	11.1 (0.0-22.2)	11.1 (0.0-22.2)	0.026	>0.999	0.465	0.388	0.108	0.086	>0.999
Symptoms	12.134	7.1 (-3.6-21.4)	7.1 (-3.6-17.9)	10.7 (-3.6-21.4)	7.1 (-3.6-21.4)	0.031	>0.999	0.531	0.804	0.059	0.090	>0.999
ADL	12.132	5.9 (0.0-16.2)	4.4 (0.0-14.7)	5.9 (0.0-17.7)	5.9 (0.0-16.2)	0.033	>0.999	0.397	>0.999	0.060	0.136	>0.999
Sport/Rec	12.107	25.0 (5.0-40.0)	25.0 (5.0-45.0)	25.0 (5.0-50.0)	30.0 (5.0-50.0)	0.015	>0.999	0.220	0.012	>0.999	>0.999	>0.999
QoL	12.120	25.0 (6.3-43.4)	25.0 (6.3-43.4)	31.3 (12.5-43.8)	31.3 (12.5-43.8)	0.001	>0.999	0.097	0.002	>0.999	0.308	>0.999
KOOS4	12.090	17.2 (4.2-30.7)	17.9 (3.7-30.1)	19.5 (5.6-32.8)	19.5 (6.3-32.4)	0.003	>0.999	0.072	0.010	0.370	0.190	>0.999
MIC												
Pain	12.130	817 (69.0)	254 (65.6)	1,789 (72.2)	5,853 (72.4)	0.004	>0.999	0.280	0.092	0.057	0.027	>0.999
Symptoms	12.134	818 (69.1)	262 (67.7)	1,785 (72.0)	5,796 (71.7)	0.089						
ADL	12.132	721 (60.9)	224 (57.9)	1,570 (63.3)	5,074 (62.8)	0.122						
Sport/Rec	12.107	760 (64.3)	266 (68.7)	1,642 (66.4)	5,574 (69.1)	0.002	0.743	>0.999	0.006	>0.999	>0.999	0.064
QoL	12.120	775 (65.5)	253 (65.4)	1,701 (68.6)	5,640 (69.9)	0.007	>0.999	0.355	0.016	>0.999	0.372	>0.999
KOOS4	12.090	784 (66.3)	256 (66.1)	1,706 (69.1)	5,688 (70.6)	0.006	>0.999	0.615	0.017	>0.999	0.360	0.824
PASS												
Pain	16.312	766 (41.4)	275 (47.8)	1,588 (45.9)	5,187 (49.8)	<0.001	0.042	0.012	<0.001	>0.999	>0.999	<0.001
Symptoms	16.314	752 (40.6)	242 (42.1)	1,532 (44.2)	4,906 (47.1)	<0.001	>0.999	0.074	<0.001	>0.999	0.124	0.022
ADL	16.311	968 (52.3)	348 (60.5)	2,038 (58.8)	6,405 (61.5)	<0.001	0.003	<0.001	<0.001	>0.999	>0.999	0.033
Sport/Rec	16.301	783 (42.3)	275 (47.8)	1,633 (47.2)	5,250 (50.4)	<0.001	0.126	0.005	<0.001	>0.999	>0.999	0.006
QoL	16.302	632 (34.2)	216 (37.6)	1,243 (35.9)	4,121 (39.6)	<0.001	0.879	>0.999	<0.001	>0.999	>0.999	<0.001
KOOS4	16.285	708 (38.3)	248 (43.1)	1,467 (42.4)	4,783 (46.0)	<0.001	0.241	0.022	<0.001	>0.999	>0.999	0.002
TF												
Pain	16.312	172 (9.3)	32 (5.6)	291 (8.4)	738 (7.1)	<0.001	0.027	>0.999	0.005	0.119	>0.999	0.063
Symptoms	16.314	311 (16.8)	70 (12.2)	462 (13.3)	1,269 (12.2)	<0.001	0.043	0.004	<0.001	>0.999	>0.999	0.446
ADL	16.311	215 (11.6)	47 (8.2)	374 (10.8)	897 (8.6)	<0.001	0.125	>0.999	<0.001	0.390	>0.999	<0.001
Sport/Rec	16.301	297 (16.1)	59 (10.3)	451 (13.0)	1180 (11.3)	<0.001	0.003	0.017	<0.001	0.403	>0.999	0.047
QoL	16.302	238 (12.9)	65 (11.3)	394 (11.4)	1044 (10.0)	<0.001	>0.999	0.657	0.002	>0.999	>0.999	0.155
KOOS4	16.285	219 (11.8)	44 (7.7)	333 (9.6)	906 (8.7)	<0.001	0.027	0.074	<0.001	0.850	>0.999	0.630

Data are reported as median (25th-75th percentile) or n (%). Range 0-100, worst to best. ADL, activities of daily living; HCHV, high caseload and high volume; HCLV, high caseload and low volume; KOOS, knee injury and osteoarthritis outcome score; KOOS4, average score of the KOOS subscales pain, symptoms, sport/rec and qol; LCHV, low caseload and high volume; LCLV, low caseload and low volume; MIC, minimal important change; PASS, patient acceptable symptom state; QoL, quality of life, Sport/Rec, sports and recreation; TF, treatment failure.

KOOS-data presented by clinic groups

	Overall	LCLV	LCHV	HCLV	HCHV		Bonferroni-adjusted *P* values					
	(N = 16,317)	(n = 4095)	(n = 3730)	(n = 453)	(n = 8039)	*P* value	LCLV vs LCHV	LCLV vs HCLV	LCLV vs HCHV	LCHV vs HCLV	LCHV vs HCHV	HCLV vs HCHV
Preoperative KOOS, yes (%)	12,139 (74.4)	2,488 (60.8)	2,702 (72.4)	314 (69.3)	6,635 (82.5)	<0.001	<0.001	0.002	<0.001	0.994	<0.001	<0.001
Pain	12.134	75.0 (61.1-86.1)	75.0 (61.1-86.1)	75.0 (63.9-86.1)	77.8 (63.9-88.9)	<0.001	0.087	>0.999	<0.001	>0.999	<0.001	0.014
Symptoms	12.136	67.9 (53.6-82.1)	67.9 (53.6-82.1)	67.9 (56.3-82.1)	71.4 (57.1-85.7)	<0.001	0.107	>0.999	<0.001	>0.999	<0.001	0.026
ADL	12.134	86.8 (70.6-95.6)	88.3 (72.1-95.6)	88.2 (71.7-95.6)	89.7 (75.0-97.1)	<0.001	0.113	>0.999	<0.001	>0.999	<0.001	0.061
Sport/Rec	12.116	35.0 (15.0-55.0)	35.0 (15.0-60.0)	35.0 (15.0-60.0)	40.0 (15.0-60.0)	<0.001	0.514	>0.999	<0.001	>0.999	<0.001	>0.999
QoL	12.128	31.3 (18.8-43.8)	31.3 (18.8-43.8)	31.3 (18.8-43.8)	31.3 (18.8-43.8)	<0.001	0.056	>0.999	<0.001	>0.999	0.056	0.848
KOOS4	12.109	51.5 (40.2-63.8)	53.2 (40.2-65.8)	53.1 (41.8-64.9)	55.6 (42.7-67.7)	<0.001	0.055	>0.999	<0.001	>0.999	<0.001	0.224
Two-year KOOS												
Pain	16.312	88.9 (75.0-97.2)	88.9 (77.8-97.2)	88.9 (75.0-97.2)	91.7 (77.8-97.2)	<0.001	0.192	>0.999	<0.001	>0.999	<0.001	0.026
Symptoms	16.314	82.1 (64.3-92.9)	82.1 (67.9-92.9)	78.6 (64.3-89.3)	82.1 (67.9-92.9)	<0.001	0.065	>0.999	<0.001	0.087	0.006	<0.001
ADL	16.311	97.1 (88.2-100.0)	97.1 (88.2-100.0)	97.1 (86.8-100.0)	97.1 (89.7-100.0)	<0.001	0.432	>0.999	<0.001	>0.999	<0.001	0.032
Sport/Rec	16.301	70.0 (45.0-85.0)	70.0 (45.0-85.0)	70.0 (40.0-85.0)	75.0 (50.0-90.0)	<0.001	0.089	>0.999	<0.001	>0.999	<0.001	0.017
QoL	16.302	62.5 (43.8-75.0)	62.5 (43.8-81.3)	62.5 (43.8-81.3)	62.5 (43.8-81.3)	<0.001	0.008	>0.999	<0.001	>0.999	0.023	0.771
KOOS4	16.285	73.9 (57.7-86.6)	75.7 (60.1-87.1)	74.5 (56.4-86.8)	77.4 (61.4-88.7)	<0.001	0.023	>0.999	<0.001	>0.999	<0.001	0.019
KOOS difference												
Pain	12.130	11.1 (0.0-22.2)	11.1 (0.0-22.2)	11.1 (0.0-22.2)	8.3 (0.0-19.4)	0.003	0.864	>0.999	0.003	>0.999	0.489	0.674
Symptoms	12.134	10.7 (-3.6-25.0)	10.7 (-3.6-25.0)	7.1 (-3.6-21.4)	7.1 (-3.6-21.4)	<0.001	>0.999	>0.999	<0.001	>0.999	0.008	>0.999
ADL	12.132	5.9 (0.0-17.7)	5.9 (0.0-16.9)	7.4 (0.0-17.7)	4.4 (0.0-14.7)	<0.001	>0.999	>0.999	<0.001	>0.999	0.028	0.262
Sport/Rec	12.107	25.0 (5.0-50.0)	25.0 (5.0-50.0)	25.0 (5.0-50.0)	30.0 (5.0-50.0)	0.599						
QoL	12.120	31.3 (12.5-43.8)	31.3 (12.5-43.8)	31.3 (12.5-43.8)	31.3 (12.5-43.8)	0.834						
KOOS4	12.090	19.5 (6.2-33.2)	19.3 (5.9-32.4)	18.5 (5.7-32.0)	19.1 (5.7-31.8)	0.360						
MIC												
Pain	12.130	1,815 (73.0)	1,946 (72.1)	232 (73.9)	4,720 (71.2)	0.286						
Symptoms	12.134	1,800 (72.3)	1,958 (72.5)	226 (72.0)	4,677 (70.5)	0.142						
ADL	12.132	1,589 (63.9)	1,731 (64.1)	203 (64.6)	4,066 (61.3)	0.024	>0.999	>0.999	0.155	>0.999	0.076	>0.999
Sport/Rec	12.107	1,695 (68.2)	1,840 (68.2)	202 (64.3)	4,505 (68.2)	0.556						
QoL	12.120	1,712 (68.9)	1,865 (69.1)	220 (70.1)	4,572 (69.0)	0.980						
KOOS4	12.090	1,732 (69.7)	1,894 (70.3)	215 (68.5)	4,593 (69.6)	0.863						
PASS												
Pain	16.312	1,799 (43.9)	1,747 (46.8)	192 (42.4)	4,078 (50.8)	<0.001	0.065	>0.999	<0.001	0.484	<0.001	0.004
Symptoms	16.314	1,764 (43.1)	1,654 (44.4)	183 (40.4)	3,831 (47.7)	<0.001	>0.999	>0.999	<0.001	0.654	0.006	0.016
ADL	16.311	2,311 (56.5)	2,187 (58.6)	260 (57.4)	5,001 (62.2)	<0.001	0.326	>0.999	<0.001	>0.999	0.001	0.249
Sport/Rec	16.301	1,835 (44.8)	1,784 (47.9)	205 (45.3)	4,117 (51.3)	<0.001	0.047	>0.999	<0.001	>0.999	0.004	0.082
QoL	16.302	1,434 (35.1)	1,405 (37.7)	175 (38.6)	3,198 (39.8)	<0.001	0.092	0.801	<0.001	>0.999	0.170	>0.999
KOOS4	16.285	1,659 (40.6)	1,613 (43.3)	184 (40.6)	3,750 (46.8)	<0.001	0.084	>0.999	<0.001	>0.999	0.003	0.070
TF												
Pain	16.312	352 (8.6)	286 (7.7)	37 (8.2)	558 (6.9)	0.012	0.820	>0.999	0.008	>0.999	0.998	>0.999
Symptoms	16.314	592 (14.5)	476 (12.8)	68 (15.0)	976 (12.1)	0.002	0.193	>0.999	0.002	>0.999	>0.999	0.464
ADL	16.311	440 (10.8)	355 (9.5)	55 (12.1)	683 (8.5)	<0.001	0.434	>0.999	<0.001	0.475	0.413	0.060
Sport/Rec	16.301	568 (13.9)	466 (12.5)	67 (14.8)	886 (11.0)	<0.001	0.459	>0.999	<0.001	>0.999	0.127	0.105
QoL	16.302	493 (12.1)	389 (10.4)	53 (11.7)	806 (10.0)	0.007	0.159	>0.999	0.005	>0.999	>0.999	>0.999
KOOS4	16.285	437 (10.7)	350 (9.4)	50 (11.0)	665 (8.3)	<0.001	0.360	>0.999	<0.001	>0.999	0.286	0.273

Data are reported as median (25th-75th percentile) or n (%). Range 0-100, worst to best. ADL, activities of daily living; HCHV, high caseload and high volume; HCLV, high caseload and low volume; KOOS, knee injury and osteoarthritis outcome score; KOOS4, average score of the KOOS subscales pain, symptoms, sport/rec and qol; LCHV, low caseload and high volume; LCLV, low caseload and low volume; MIC, minimal important change; PASS, patient acceptable symptom state; QoL, quality of life, Sport/Rec, sports and recreation; TF, treatment failure.
