# Supplementary material for: Influence of Surgeon Experience and Clinic Volume on Subjective Knee Function and Revision Rates in Primary ACL Reconstruction: A Study from the Swedish National Knee Ligament Registry
Source: Orthop J Sports Med. 2024 Mar 11;12(3):23259671241233695. doi: 10.1177/23259671241233695 (PMC10929050; doi:10.1177/23259671241233695)
Supplement: sj-pdf-3-ojs-10.1177_23259671241233695 – Supplemental material for Influence of Surgeon Experience and Clinic Volume on Subjective Knee Function and Revision Rates in Primary ACL Reconstruction: A Study from the Swedish National Knee Ligament Registry [file sj-pdf-3-ojs-10.1177_23259671241233695.pdf]

Supplemental Material 3: Responders of 2-year KOOS

Proportion of two-year KOOS responders by surgeon volume groups

	Overall (N = 34,126)	LCLV (n = 3868)	LCHV (n = 1200)	HCLV (n = 7320)	HCHV (n = 21,738)	*P* value	Bonferroni-adjusted *P* values					
							LCLV vs LCHV	LCLV vs HCLV	LCLV vs HCHV	LCHV vs HCLV	LCHV vs HCHV	HCLV vs HCHV
N responders (%)	16,317 (47.8)	1850 (47.8)	575 (47.9)	3466 (47.3)	10,426 (48.0)	0.843	—	—	—	—	—	—

Dashes indicate areas not applicable. HCHV, high caseload and high volume; HCLV, high caseload and low volume; LCHV, low caseload and high volume; LCLV, low caseload and low volume

Proportion of two-year KOOS responders by clinic volume groups

	Overall (N = 34,126)	LCLV (n = 8564)	LCHV (n = 7752)	HCLV (n = 1135)	HCHV (n = 16,675)	*P* value	Bonferroni-adjusted *P* values					
							LCLV vs LCHV	LCLV vs HCLV	LCLV vs HCHV	LCHV vs HCLV	LCHV vs HCHV	HCLV vs HCHV
N responders (%)	16,317 (47.8)	4095 (47.8)	3730 (48.1)	453 (39.9)	8039 (48.2)	<0.001	>0.999	<0.001	>0.999	<0.001	>0.999	<0.001

HCHV, high caseload and high volume; HCLV, high caseload and low volume; LCHV, low caseload and high volume; LCLV, low caseload and low volume
