# Supplementary material for: Influence of Surgeon Experience and Clinic Volume on Subjective Knee Function and Revision Rates in Primary ACL Reconstruction: A Study from the Swedish National Knee Ligament Registry
Source: Orthop J Sports Med. 2024 Mar 11;12(3):23259671241233695. doi: 10.1177/23259671241233695 (PMC10929050; doi:10.1177/23259671241233695)
Supplement: sj-pdf-4-ojs-10.1177_23259671241233695 – Supplemental material for Influence of Surgeon Experience and Clinic Volume on Subjective Knee Function and Revision Rates in Primary ACL Reconstruction: A Study from the Swedish National Knee Ligament Registry [file sj-pdf-4-ojs-10.1177_23259671241233695.pdf]

Supplemental Material 4: Results of logistic regression analyses

Variables influencing the odds of MIC Pain
(n patients = 8,317)

	OR	95% CI	*P* value
Age at surgery	1.024	1.019-1.029	<0.001
Female sex	0.834	0.749-0.930	0.001
Time to surgery	0.998	0.996-0.999	0.009
BMI	0.967	0.950-0.983	<0.001
ACL graft			
PT	0.729	0.562-0.946	0.017
QT	0.627	0.431-0.911	0.014
HT	Ref		
Surgeon experience			
LCLV	0.731	0.610-0.876	<0.001
LCHV	0.785	0.587-1.051	0.104
HCLV	0.922	0.807-1.052	0.228
HCHV	Ref		
Preop KOOS Pain	0.938	0.935-0.942	<0.001

Variables influencing the odds of MIC ADL
(n patients = 8,320)

	OR	95% CI	*P* value
Age at surgery	1.020	1.014-1.025	<0.001
Female sex	1.116	1.003-1.243	0.045
Time to surgery	0.997	0.996-0.999	<0.001
BMI	0.978	0.962-0.995	0.013
Meniscal injury	1.152	1.035-1.282	0.009
Chondral injury	0.843	0.743-0.957	0.008
Periop complications	0.637	0.413-0.983	0.042
Surgeon experience			
LCLV	0.777	0.646.0.934	0.007
LCHV	0.855	0.636-1.149	0.300
HCLV	0.965	0.846-1.101	0.594
HCHV	Ref		
Preop KOOS ADL	0.899	0.894-0.904	<0.001

Variables influencing the odds of MIC Symptoms
(n patients = 8,318)

	OR	95% CI	*P* value
Age at surgery	1.025	1.019-1.030	<0.001
Female sex	0.903	0.811-1.005	0.060
BMI	0.967	0.951-0.983	<0.001
Operating time	0.997	0.995-0.999	0.004
ACL graft			
PT	0.825	0.638-1.067	0.143
QT	0.680	0.470-0.982	0.040
HT	Ref		
Preop KOOS Symptoms	0.949	0.946-0.953	<0.001

Logistic regression analyses adjusted for age at surgery, sex, BMI, activity at time of injury (pivoting contact sport), meniscal injury, chondral injury, medial collateral ligament injury, time from injury to surgery (months), operating time (minutes), perioperative complications, ACL graft choice, preoperative KOOS, surgeon groups and clinic groups. Only variables influencing the odds are retained in the model (if p <0.010). Results are presented as odds ratio (OR) with 95 % confidence interval (CI). ACL, anterior cruciate ligament; ADL, activity of daily living; BMI, body mass index; HCHV, high caseload and high volume; HCLV, high caseload and low volume; HT, hamstring tendon; KOOS4, average score of the KOOS subscales pain, symptoms, sport/rec and qol; LCHV, low caseload and high volume; LCLV, low caseload and low volume; MIC, minimal important change; PASS, patient acceptable symptom state; Periop, perioperative; Preop, preoperative; PT, patellar tendon; QoL, quality of life; QT, quadriceps tendon; Sport/Rec, sports and recreation; TF, treatment failure.

Variables influencing the odds of PASS Pain
(n patients = 8,317)

	OR	95% CI	*P* value
Age at surgery	1.023	1.019-1.028	<0.001
Female sex	0.805	0.732-0.885	<0.001
BMI	0.975	0.961-0.990	<0.001
MCL injury	0.736	0.585-0.926	0.009
Chondral injury	0.827	0.742-0.921	<0.001
Periop complications	0.673	0.459-0.987	0.043
Surgeon experience			
LCLV	0.729	0.619-0.857	<0.001
LCHV	0.864	0.660-1.133	0.291
HCLV	0.865	0.771-0.972	0.014
HCHV	Ref		
Preop KOOS Symptoms	1.042	1.039-1.045	<0.001

Variables influencing the odds of PASS Symptoms
(n patients = 8,318)

	OR	95% CI	*P* value
Age at surgery	1.021	1.017-1.026	<0.001
Female sex	0.877	0.799-0.964	0.006
BMI	0.974	0.959-0.988	<0.001
Operating time	0.997	0.995-0.999	0.004
Preop KOOS Symptoms	1.035	1.033-1.038	<0.001

Variables influencing the odds of PASS ADL
(n patients = 8,320)

	OR	95% CI	*P* value
Age at surgery	1.013	1.007-1.018	<0.001
Female sex	0.844	0.762-0.934	0.001
BMI	0.942	0.928-0.957	<0.001
Pivoting contact sport	0.876	0.785-0.978	0.018
Meniscal injury	1.136	1.029-1.254	0.012
Chondral injury	0.844	0.753-0.946	0.003
Periop complications	0.579	0.395-0.849	0.005
ACL graft			
PT	0.982	0.767-1.257	0.885
QT	0.644	0.456-0.910	0.012
HT	Ref		
Surgeon experience			
LCLV	0.708	0.600-0.836	<0.001
LCHV	0.881	0.664-1.169	0.379
HCLV	0.989	0.875-1.117	0.855
HCHV	Ref		
Preop KOOS ADL	1.045	1.042-1.048	<0.001

Variables influencing the odds of PASS QoL
(n patients = 8,314)

	OR	95% CI	*P* value
Age at surgery	1.026	1.021-1.030	<0.001
Female sex	0.861	0.782-0.948	0.002
BMI	0.966	0.951-0.980	<0.001
Pivoting contact sport	0.899	0.810-0.997	0.043
Chondral injury	0.857	0.769-0.954	0.005
Periop complications	0.600	0.398-0.906	0.015
Preop KOOS QoL	1.028	1.025-1.030	<0.001

Logistic regression analyses adjusted for age at surgery, sex, BMI, activity at time of injury (pivoting contact sport), meniscal injury, chondral injury, medial collateral ligament injury, time from injury to surgery (months), operating time (minutes), perioperative complications, ACL graft choice, preoperative KOOS, surgeon groups and clinic groups. Only variables influencing the odds are retained in the model (if p <0.010). Results are presented as odds ratio (OR) with 95 % confidence interval (CI). ACL, anterior cruciate ligament; ADL, activity of daily living; BMI, body mass index; HCHV, high caseload and high volume; HCLV, high caseload and low volume; HT, hamstring tendon; KOOS4, average score of the KOOS subscales pain, symptoms, sport/rec and qol; LCHV, low caseload and high volume; LCLV, low caseload and low volume; MIC, minimal important change; PASS, patient acceptable symptom state; Periop, perioperative; Preop, preoperative; PT, patellar tendon; QoL, quality of life; QT, quadriceps tendon; Sport/Rec, sports and recreation; TF, treatment failure.

Variables influencing the odds of TF Pain
(n patients = 8,317)

	OR	95% CI	*P* value
Age at surgery	0.989	0.980-0.998	0.015
Female sex	1.310	1.081-1.587	0.006
BMI	1.030	1.002-1.058	0.039
Chondral injury	1.320	1.074-1.623	0.008
Preop KOOS Pain	0.950	0.946-0.955	<0.001

Variables influencing the odds of TF Symptoms
(n patients = 8,318)

	OR	95% CI	*P* value
Age at surgery	0.981	0.974-0.988	<0.001
Female sex	1.218	1.054-1.406	0.008
BMI	1.048	1.027-1.071	<0.001
Operating time	1.005	1.003-1.008	<0.001
Preop KOOS Symptoms	0.963	0.959-0.966	<0.001

Variables influencing the odds of TF ADL
(n patients = 8,320)

	OR	95% CI	*P* value
Age at surgery	0.991	0.983-0.999	0.032
Female sex	0.957	0.802-1.141	0.622
BMI	1.046	1.020-1.073	<0.001
Chondral injury	1.331	1.102-1.608	0.003
Preop KOOS ADL	0.953	0.950-0.957	<0.001

Variables influencing the odds of TF Sport/Rec
(n patients = 8,307)

	OR	95% CI	*P* value
Age at surgery	0.989	0.982-0.996	0.002
Female sex	1.415	1.217-1.646	<0.001
BMI	1.081	1.059-1.104	<0.001
Chondral injury	1.314	1.118-1.543	<0.001
Preop KOOS Sport/Rec	0.968	0.965-0.972	<0.001

Variables influencing the odds of TF QoL
(n patients = 8,314)

	OR	95% CI	*P* value
Age at surgery	0.967	0.959-0.974	<0.001
Female sex	1.147	0.981-1.342	0.085
BMI	1.056	1.033-1.080	<0.001
Preop KOOS Sport/Rec	0.954	0.949-0.959	<0.001

Variables influencing the odds of TF KOOS4
(n patients = 8,320)

	OR	95% CI	*P* value
Age at surgery	0.981	0.973-0.989	<0.001
Female sex	1.225	1.030-1.456	0.021
BMI	1.059	1.033-1.084	<0.001
Chondral injury	1.272	1.055-1.533	0.012
Preop KOOS4	0.946	0.940-0.951	<0.001

Logistic regression analyses adjusted for age at surgery, sex, BMI, activity at time of injury (pivoting contact sport), meniscal injury, chondral injury, medial collateral ligament injury, time from injury to surgery (months), operating time (minutes), perioperative complications, ACL graft choice, preoperative KOOS, surgeon groups and clinic groups. Only variables influencing the odds are retained in the model (if p <0.010). Results are presented as odds ratio (OR) with 95 % confidence interval (CI). ACL, anterior cruciate ligament; ADL, activity of daily living; BMI, body mass index; HCHV, high caseload and high volume; HCLV, high caseload and low volume; HT, hamstring tendon; KOOS4, average score of the KOOS subscales pain, symptoms, sport/rec and qol; LCHV, low caseload and high volume; LCLV, low caseload and low volume; MIC, minimal important change; PASS, patient acceptable symptom state; Periop, perioperative; Preop, preoperative; PT, patellar tendon; QoL, quality of life; QT, quadriceps tendon; Sport/Rec, sports and recreation; TF, treatment failure.
